# Relapsing cellulitis associated with *Campylobacter coli* bacteremia in a Good’s syndrome patient: a case report

**DOI:** 10.1186/s12879-022-07324-3

**Published:** 2022-04-09

**Authors:** Lei Jiang, Jia Gao, Pu Wang, Yuliang Liu

**Affiliations:** 1 Department of Respiratory Medicine, The First Affiliated Hospital of Chongqing Medical University, Chongqing, China; 2 Department of Radiology, The First Affiliated Hospital of Chongqing Medical University, Chongqing, China

**Keywords:** *Campylobacter coli*, Bacteremia, Cellulitis, Case report

## Abstract

**Background:**

Good’s syndrome (GS) is characterized by immunodeficiency, and patients diagnosed with GS are susceptible to infection or even bacteremia, which is the most evident complication. *Campylobacter coli* (*C*. *coli*) rarely causes bacteremia or extraintestinal infection. We report herein a case with GS in which right leg cellulitis associated with *C. coli* bacteremia occurred three times over one and a half years.

**Case presentation:**

A 41-year-old Chinese male with GS was diagnosed with *C. coli* infection. He presented with swelling and redness of right lower leg and developed bacteremia due to *C*. *coli* repeatedly. Bacteremia was confirmed by bacteriological examination. Adding long-term oral antibiotic treatment with amoxicillin/clavulanate potassium and gentamicin following intravenous meropenem and amikacin was very effective. The blood cultures became negative and the patient has been free from any symptoms encountered for more than one year without relapse of bacteremia.

**Conclusions:**

Patients with GS and their physicians should carefully consider the antibacterial treatment options against *C. coli* bacteremia. Combined anti-infective therapy involving aminoglycoside is preferred in the treatment of *C. coli* bacteremia in GS patients.

## Background

Good’s syndrome mainly occurs in 40 to 60-year-old adults, with a prevalence of 1/700,000 [1]. It is characterized by the presence of thymoma associated with hypogammaglobulinemia, low or absent peripheral B cells and defects in cell-mediated immunity [2]. The association of thymoma and immunodeficiency has not been well explained, and the pathophysiological mechanism of GS remains unclear. Lack of B cells, T cells and immunoglobublin seriously impairs the immunity and renders the patient highly susceptible to infection.

Infection is the leading cause of death in GS patients. Sinopulmonary tract, gastrointestinal system, skin and soft tissue are most likely to be affected by pathogens. The most frequent causative bacterium, fungus and virus are *Pseudomonas spp*., *Candida spp*., and cytomegalovirus. *Campylobacter spp.* is the fourth most common bacterial pathogen in GS patients [3]. *Campylobacter coli* (*C. coli*) usually causes bacterial diarrhea, rarely leads to bacteremia or extraintestinal complications in immunologically normal hosts [4]. However, in immunocompromised patients, these pathogens occasionally develop extraintestinal infection.

Here, we report the first case of GS who suffered from relapsing *C. coli* bacteremia with leg cellulitis. We depict the clinical course and explore the pathogenesis and treatment.

## Case presentation

**Fig. 1:**
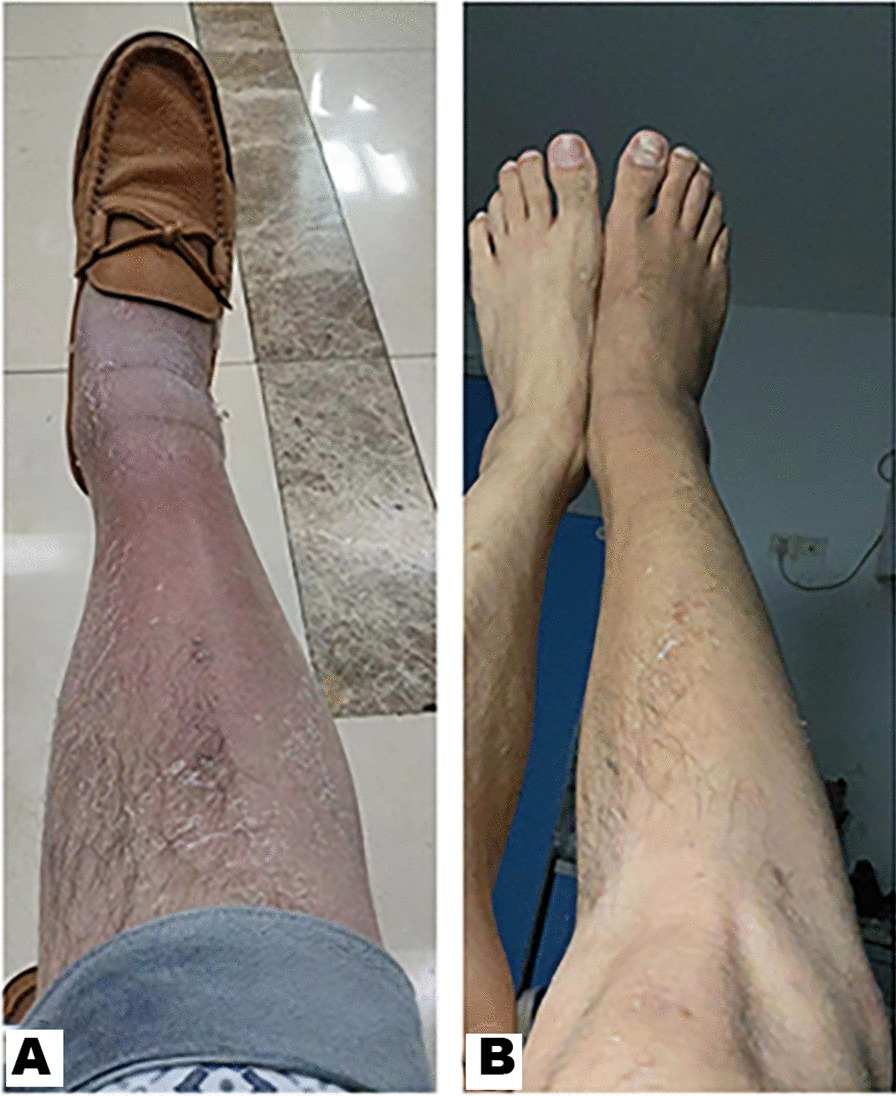
Analysis of Vital signs of the right lower leg. **A** On his admission, his right lower leg was red and swollen obviously. **B** After effective treatment, his right leg became nearly normal

A 41-year-old man was admitted to our hospital presenting with swelling and redness of right lower leg for half a year (Fig. 1A). On admission, his body temperature was 36.9 ℃, pulse rate was 99 per min, blood pressure was 106/75 mmHg, respiratory rate was 21 per min, and he was conscious. He had suffered from pneumonia several times, hepatitis B and tuberculosis. Seven years ago, he had a chest computed tomography (CT) performed on him suggesting a thymoma and therefore he underwent thymectomy. After the thymoma resection, he had been in health without recurrence, and received no medical treatment. His family history was unremarkable. He had not contact with any animals commonly infected with *C. coli*, including chicken, cattle, pigs, etc. He had no history of eating uncooked chicken, beef, or pork neither. He had not complained of any abdominal symptoms including abdominal pain and diarrhea.

A cellular immunoassay was performed during hospitalization. Results revealed markedly reduced lymphocytes with 552 total cells/μL (normal lower value 1000 cells/μL), CD4+ T cells = 104 cells/μL (normal lower value 580 cells/μL), CD8+ T cells = 333 cells/μL (normal lower value 200 cells/μL), an inverted CD4/CD8 T-cell ratio of 0.31 (normal value 1–1.5), CD19+ CD20+ B-lymphocytes = 0 cells/μL (normal lower value 80 cells/μL), CD3+ CD16+ CD56+ natural killer (NK) lymphocytes = 8 cells/μL (normal value 40–600 cells/μL). HIV test showed negative and a severe hypogammaglobulinemia existed, with IgG 253 mg/dL (normal value 751–1560 mg/dL), IgA 12 mg/dL (normal value 82–453 mg/dL), and IgM 4 mg/dL (normal value, 46–304 mg/dL). On the basis of (1) past history of thymoma, (2) B lymphocyte deficiency and CD4 + T lymphopenia, (3) hypogammaglobulinemia, (4) repeated infections, he was reasonably diagnosed as having GS.

The patient was treated with intravenous cefoperazone/sulbactam (3.0 g, every 8 h) and teicoplanin (0.4 g, per day) for 1 week. Because *C. coli* was isolated from the blood specimen at the time of admission, the therapy was changed to intravenous administration of imipenem/cilastatin (1.0 g, every 8 h) and oral azithromycin (0.5 g, per day), which are recommended as empiric agents for *Campylobacter* bacteremia [5]. His symptoms and the inflammation improved. 11 days later, the therapy was changed to oral administration of azithromycin for 4 weeks only. However, the cellulitis on his right lower leg relapsed and blood culture yielded *C. coli* again. Then intravenous imipenem/cilastatin for 2 weeks was re-added to the treatment. The cellulitis was resolved (Fig. 1B) and blood culture became negative shortly. The patient was discharged on the 59th hospital day. Intravenous meropenem (1.0 g, every 8 h) and oral azithromycin for one month were administered in the local hospital near his home.

However, the *C. coli* bacteremia accompanied by leg cellulitis relapsed two times, 4 and 7 months later. Intravenous administration cefoperazone/sulbactam and etimicin (0.3 g, per day) for 3 weeks were effective for the bacteremia at the 1st relapse. The patient was discharged after the therapy was changed from intravenous administration of cefoperazone/sulbactam and etimicin to oral administration of minocycline (0.1 g, twice a day), clarithromycin (0.25 g, twice a day) and faropenem (0.15 g, 3 times a day) for 1 month. Intravenous meropenem (2.0 g, every 8 h) and amikacin (0.4 g, per day) for 1 month were effective for the bacteremia at the 2nd relapse. Due to hypogammaglobulinemia, immunoglobulin (Ig)-replacement therapy (10 g, per day for 2 weeks) was provided in this period. IgG level was markedly elevated by immunoglobulin infusion. However, IgM and IgA levels were almost unaffected. IgA fluctuated between 12 and 17 mg/dL and IgM fluctuated between 4 and 11 mg/dL. The patient was discharged after the therapy was changed from intravenous administration of meropenem and amikacin to oral administration of amoxicillin/clavulanate potassium (0.457 g, four times a day) and gentamicin (0.12 g, four times a day) for 3 months.

A painless gastroscopic examination revealed chronic superficial antral gastritis while colonoscopic examination showed no abnormality. A whole-body computed tomography (CT) scan provided no valuable evidence of visceral infection, and the transthoracic echocardiography did not suggest infective endocarditis. Magnetic resonance imaging (MRI) of his lower legs showed high intensity at the subcutaneous tissues on long T1 and T2 inversion recovery, but an abscess or osteomyelitis formation was not indicated. Although the fecal specimen was obtained repeatedly, the stool cultures were negative for *C. coli*.

The patient has been regularly followed up in our outpatient department. He has been feeling well and living a normal life for about one year without relapse of bacteremia. No adverse and unanticipated events were reported by him.

## Discussion and conclusions

*Campylobacter coli* bacteremia has been reported mainly in hypogammaglobulinemia patients, however, rarely mentioned in GS patients. We presented a case of recurrent episodes of *C. coli* bacteremia with cellulitis in an adult GS patient.

Over 1 year, the patient had at least three episodes of *C. coli* bacteremia, with a redness that intermittently emerged on his lower limb, which was caused by immunodeficiency in relation to GS. In GS, several aspects of immunodeficiency may occur in addition to hypogammaglobulinemia, including low circulating B lymphocytes, CD4+ T-cell lymphopenia and reversal CD4+ to CD8+ ratio [2]. As a result, the patient’s humoral and cellular response were unable to deal with the pathogens.

*Campylobacter coli* is a gram-negative bacillus that can infect the intestinal tract of cattle and may be the causative agent of enteritis in humans. *C. coli* mainly causes colitis and rarely bacteremia except in immunocompromised patients. It has been reported that *C. coli* can be detected in the biopsy simples from the intestinal mucosa in an immunocompromised host [6]. Intestinal tissue invasion of *C. coli* may be one of the pathogenetic mechanisms of recurring infection in GS patients. In addition, up to 80% of human bacterial infections are caused by bacterial biofilms [7]. *C. coli* may have the ability to form biofilms which contribute to antimicrobial resistance on mucosal surfaces of the intestine [8, 9].

Gut immunity via IgA which exists in the gastrointestinal mucosa may play a vital role in the defense against *C. coli* infection. The extremely low levels of IgA make GS patients more susceptible to *C. coli* infection and impede elimination of pathogens in the gut. Therefore, GS patients are liable to suffer from the latent intestinal infection with *C. coli* leading to the recurrent bacteremia [10, 11]^.^ Ig-replacement therapy in this case exerted little benefit in the treatment of *C. coli* infection in spite of IgG levels elevated obviously. *C. coli* bacteremia has been reported to occur even in the presence of high serum IgG levels in X-linked agammaglobulinemia patients (XLA) [12, 13], because IgA is not provided with Ig-replacement therapy. Another way to boost gut immunity may need to be explored.

In our case, *C. coli* was isolated from blood culture with an exacerbation of the patient’s cellulitis. We believe that the microorganism was related to cellulitis, although *C. coli* was not directly detected in the cellulitis lesion [14]. The occurrent rate of cellulitis varies from 3 to 20% and is extremely common in the lower limbs with multiple episodes of bacteremia [15], which was also seen in our patient. The pathophysiology for development of the cellulitis remains to be investigated further.

Although erythromycin has been firstly used to treat *Campylobactor* infection, the prevalence of erythromycin-resistant *Campylobacter* is evidently increasing [16]. *C. coli* has a rate of azithromycin resistance of 11% in 2020. Azithromycin used in our patient failed to eradicate *C. coli* in the blood. Inhibition of bacterial biofilm formation may be helpful for overcoming antimicrobial resistance and eliminating *C. coli* in the body [17]. Carbapenem is recommended for empirical treatment of *Campylobacter* bacteremia, however, *C. coli* has been shown elevated minimum inhibitory concentrations to meropenem in a patient with XLA due to overuse [12]. If refractory *Campylobacter* infection or bacteremia occurs, combination therapy may be an appropriate approach [6, 14]. Additionally, aminoglycosides may be beneficial in the treatment of multidrug-resistant *Campylobacter* infections [12]. The incidence of aminoglycoside-resistant bacteria is about 3% [18], therefore, aminoglycoside is recommended especially for the case of severe infections. Oral gentamicin in our patient has been shown effective for the remaining follow-up time.

Persistent carriage of *Campylobacter* in the bowel is considered the major source of bloodstream infections [19]. In our case, the oral administration of amoxicillin/clavulanate potassium and gentamicin proved successful in the treatment, promoting eradication of *C. coli* in the intestinal tract and blood, although *C. coli* in fecal was negative all the time.

In conclusion, GS should be alerted for adult patients with a previous history of thymoma and recurrent systemic campylobacteriosis. Combined antibacterial regimen is effective in these patients with extraintestinal infection of *C. coli*. Aminoglycoside might be a key component of the combined treatment.

## Data Availability

The data that support the findings of this study are available from the corresponding author upon reasonable request.

## Abbreviations

GS: Good’s syndrome
*C. coli*: *Campylobacter coli*
CT: Computed tomography
MRI: Magnetic resonance imaging
Ig: Immunoglobulin
XLA: X-linked agammaglobulinemia
